# Clinical Lecture on Diseases of Women

**Published:** 1883-05

**Authors:** T. Gaillard Thomas


CLINICAL LECTURE ON THE DISEASES OF WOMEN. Delivered at the College of Physicians and Surgeons, New York. By PROF. T. GAILLARD THOMAS.

### INFANTILE LEUCORRHŒA.

GENTLEMEN: The little girl, nine years old, whom I first bring before you, is suffering from a very profuse leucorrhœa, which, her mother informs me, she has been unable to cure by any of the remedies which she has employed, and which has now lasted for two months. I, of course, made a vaginal examination, and, on separating the labia, I found that the whole vulva was about the color of red flannel, and bathed with a copious leucorrhœal discharge. The meatus urinarius was also seen to be in the same condition, and urethritis has, no doubt, been set up by the spreading of the irritation. If it had been necessary, I could have introduced a small glass speculum into the vagina; but this was not required to make a diagnosis, as I saw exactly what was the matter without resorting to this.

Not infrequently mothers will bring their little girls to you in this condition, and they will sometimes be in a state of great agitation, because they are afraid the trouble has been the result of injury done the children. There is ordinarily no reason whatever to suspect anything of the kind, and you can at once quiet the anxious mother's mind. The affection is a perfectly simple one, and is perfectly curable also. What is it, then? It is generally known as infantile leucorrhœa; but infantile vaginitis would be a better term for it.

Now as to its causes. One of the most frequent of these is neglect of hygienic precautions. There is generally no intentional neglect on the part of the mother or nurse; but, on account of the undeveloped condition of the part, an accumulation of hardened secretion sometimes collects in the same way as that which not infrequently gives rise to balanitis in the male child. Another common cause is the depreciated condition of the child's system, such as that due to spanæmia, in which all the mucous membranes are apt to become more or less affected. Thus, there is often gastric and intestinal, as well as nasal catarrh. A third cause that may be mentioned is reflex influence from the rectum. The cause of the irritation in the rectum is usually ascarides, and an afflux of blood to the part is caused by the itching and irritation.

In some instances, the ascarides, by getting into the vagina itself, are the direct cause of the trouble. The prognosis of this affection is, that it can be cured at once if it is properly treated.

In the treatment, the first thing to do is to see if there are any worms present, and if so (or there is any reason to suspect that such is the case), use an injection of warm salt water, as this form of ascaris (*the ascaris vermicularis*), as well as others, is unfavorably affected by salt. The next thing to do is to get the child's general system in the best condition possible by appropriate food, iron, vegetable tonics, and the hypophosphites. It is better to depend on nourishing diet, however, than on medicinal agents. If after the worms have been gotten rid of the vaginal irritation and discharge should continue, or if no worms should be found to be present, local treatment will be required. The vagina should be thoroughly washed out by means of a syringe provided with a small nozzle, which ought to be well oiled before being introduced. In order that the canal may be perfectly cleansed, the child should be placed upon the back. In some cases the mere removal of the accumulated secretion, which is a constant source of irritation, is all that is necessary; but if the trouble has gone on for some time, this may not be sufficient. Something further is then needed, and one of the best applications to use is the old-fashioned black wash (calomel and lime-water) in the strength of one ounce to the pint of water. Before using this (which should be done twice a day), an injection of simple warm water should be made. I have never yet seen a case of infantile leucorrhœa that could not be cured by such treatment as this; so that there is no necessity of resorting to astringents and nitrate of silver, which may perhaps do harm. If it is adopted here, I have no doubt that in less than two weeks this child will be entirely well.

But there is one mistake which is apt to be made by the physician in these cases, on account of which a much longer time may be required for a case than is at all necessary, and that is, the failure on his part to show the mother or nurse how to introduce the nozzle of the syringe properly. Mothers, unless they are especially instructed in regard to this point, never carry the nozzle more than an eighth of an inch up into the vagina, and as it is above this that the degenerating pus is found, there will be no improvement, simply because the injections fail to reach the real source of trouble. It is not enough even to show the mother how to use the syringe, but you should also watch her do it, and see that the upper part of the vagina is reached. In a child of this age, the rectal tube of a Davidson syringe should be employed.

### RECURRING ATTACKS OF PELVIC PERITONITIS IN CONNECTION WITH OVARITIS AND SALPINGITIS.

Our next patient is Mrs. Mary H., a native of the United States, and 33 years of age, who has been married for ten years. She has had two children, and the youngest is now 7 years old. She says she got quite well after the birth of her last child; but has been complaining for more than six years. She nursed the child for about seven months when her menses came on, and since that time she has never been well. This is not an uncommon history; the trouble not commencing at child-birth, but with the resumption of ovulation. Since she has stopped nursing the child, she says she has had constant pain in the lower part of the abdomen, and twice she has been very much bloated. The first time that this bloating occurred, she did not suffer much pain with it; but the second time it was accompanied with very severe pain, and she was confined to bed for a time. The second attack lasted for about two months. In regard to her menstrual periods, she says she does not have much more pain than usual at that time, but that she has considerable pain for three or four days before the flow, and that it is somewhat relieved by the latter. She sometimes has backache in addition to the constant abdominal pain, but is always able to attend to her honsehold duties.

When a vaginal examination was made in this case, it was found that the uterus was in its normal position, and, on mapping it out, by means of conjoined manipulation, that there was more or less fixation of the organ. On either side of the uterus was discovered a mass which was tender under pressure, and which extended outwards. The fixation of the uterus indicates with certainty that the patient has had pelvic peritonitis; and there can be little doubt that the two attacks of which she has spoken were of this nature. The bloating which she mentioned, and which she thought was dropsy, was in all probability the tympanites which so frequently accompanies pelvic peritonitis.

What is the diagnosis here? All the gentlemen who have examined the case, agree with me in believing that both the fallopian tubes, as well as both the ovaries, are enlarged since these parts can be mapped out with unusual ease in this instance. It is altogether probable, therefore, that after she ceased nursing her child, she became affected with chronic ovaritis and chronic salpingitis. Mr. Lawson Tait, the distinguished English surgeon, has recently pointed out the fact that repeated attacks of pelvic peritonitis occur as a result of this condition of the ovaries and tubes. Up to the time that Mr. Tait's paper appeared in the *British Medical Journal* last summer, I had never had my attention called to this point, and consequently my experience has not yet been very extensive in regard to it; but I have already seen enough to convince me that he is quite right in attributing these recurring attacks of pelvic peritonitis to the escape of irritating fluid from the inflamed tubes into the peritoneum. In the present instance, the first attack of peritonitis was evidently very light; but the second one seems to have been considerably more severe. Although the tubal enlargements can apparently be made out with unusual clearness in this case, the diagnosis, you must understand, is not a positive one.

I have not operated in five cases where the same diagnosis was made, but they were all cases in which the patient's sufferings had become intolerable. The fifth operation was performed only four days ago. The patient was completely bed-ridden, and had become so addicted to the use of opium that she required no less than ten grains of sulphate of morphia by hypodermic injection each day. Sometimes she has taken as much as sixteen grains in the twenty-four hours. To-day, for the first time, her allowance of the drug has been diminished. Her ovaries and fallopian tubes, I may say in passing, were found to be in typical condition described by Tait.

In the case now before you the ovaries and tubes are, no doubt, in very much tha same state; and yet this is a case in which I certainly would not recommend the operation for their removal. The reason is, that this woman does not suffer to such an extent, in my opinion, as to justify a resort to so radical a procedure. I cannot impress upon you too strongly the fact that the dangers of this operation are very great: and the great fault I have to find with Mr. Tait, is that he makes too light of them altogether. I cannot believe that the high standard of success which he has thus far maintained, will be kept up in the future. In the five cases which I have now operated upon myself, there is not one patient to whom death would not have been a welcome relief from her sufferings. A woman who is in such a deplorable condition as all these even before this operation, is worse than a leper, and everybody who has anything to do with her case soon gets sick of it. Such patients almost invariably become opium-eaters. The present case is fortunately not of this desperate character. The dividing line must be drawn between suitable and unsuitable cases for operation; and nothing would induce me to operate here, as long as she remains no worse than she is now. She does not suffer enough, and her life is by no means miserable enough, to render it proper to subject her to the dangers of such an operation. Saturday, however, I saw a case in which I would not hesitate an instant to operate, beeause the poor woman had had so many attacks of inflammation, and was so utterly wretched in every way.

Here, instead of an operation, I would recommend such meas ures as the repeated painting of the roof of the vagina with compound tincture of iodine, the external application of blisters or tincture of iodine, and the continued use of galvanism and the hot-water douche.

SUBINVOLUTION AND ANTEFLEXION IN CONNECTION WITH LACERATION OF THE CERVIX UTERI.

Our last patient to-day is Mrs. Mary P——, 24 years of age, and a native of the United States. She has had one child and three miscarriages, the last of which occurred one year ago. She says she has been complaining for the last three or four months, and has suffered from severe pain in the back and lower part of the abdomen. At her monthly periods, which last for a week, she flows very profusely. She also suffers a great deal of pain at the time of menstrutation, and would go to bed if it were possible for her to do so. Between her periods she has a profuse leucorrhœa. All the miscarriages, it seems, have occurred since the birth of her living child, and in answer to closer questioning she tells me that the menorrhagia has really continued ever since her baby was born, but has increased of late. The pains also she has had for a long time, but it is only within the last three or four months that they have become so severe. Finally, she says that there has been no change in her condition of life which would account for the aggravation of symptoms of which she has spoken.

When I made a physical examination in this case, the moment the finger was passed up into the vagina, it was found that the uterus was tilted forward to a marked extent, and that the cervix was badly lacerated on both sides. The uterus was then found to be not simply anteverted, but in a state of anteflexion, and was also both large and tender. The ovaries were, apparently, altogether normal. Such a condition having been found, the most of the patient's symptoms are already accounted for. When the child was born the cervix uteri was lacerated, and, no doubt, in consequence of this, involution was interferred with. The menorrhagia depends on the abnormal condition of the lining mucous membrane of the uterus, which was in all probability affected by subinvolution. The diagnosis of the case is, therefore, *first*, laceration of the cervix ; *second*, subinvolution of the uterus ; *third*, anteflexion of the uterus, in consequence of the subinvolution ; and, *fourth*, fungoid degeneration of the endometrium. Some European writers call this latter condition *endo-metritis polyposa*, but the name is not a correct one.

In order to be properly treated, this patient should be admitted to the Woman's Hospital. She should be kept quiet for a week, have her bowels regulated, and be treated with hot vaginal injections. A Sims' speculum should then be introduced, and by means of a copper wire curette, such as I now show you, the whole endometrium should be scraped, in order to remove all the little polypoid excrescences with which it is covered. After that she should remain in bed for three or four days, the hot vaginal injections being kept up in the meanwhile, and then an operation for the repair of the lacerated cervix should be performed. The sutures could be removed in about ten days, and the next thing would be to restore the uterus to its proper position, in which it would probably remain without the use of a pessary. The patient could then be discharged, and I believe that she would soon be entirely well.

You observe that the woman is very pale and weak from loss of blood ; but as long as this condition of menorrhagia continues, it is adding fuel to the flames to give her iron or quinia. Iron, I have found, is the very worst thing that you can give such a patient, and this is a point which I trust you will not lose sight of. As to the leucorrhœa, that will no doubt disappear as soon as her system, freed from the constant drain from which it now suffers, has regained its normal strength and tone.—*Medical and Surgical Reporter.*

